# Melanoma cell-secreted exosomal miR-155-5p induce proangiogenic switch of cancer-associated fibroblasts via SOCS1/JAK2/STAT3 signaling pathway

**DOI:** 10.1186/s13046-018-0911-3

**Published:** 2018-10-03

**Authors:** Xiaocheng Zhou, Tinglin Yan, Chunming Huang, Zhi Xu, Lin Wang, Erhui Jiang, Hui Wang, Yang Chen, Ke Liu, Zhe Shao, Zhengjun Shang

**Affiliations:** 1 The State Key Laboratory Breeding Base of Basic Science of Stomatology (Hubei-MOST) & Key Laboratory for Oral Biomedicine Ministry of Education, Wuhan University, Wuhan, China; 2 Center of Stomatology, Tongji Hospital, Tongji Medical College, Huazhong University of Science and Technology, Wuhan, China; 3 Department of Stomatology, Union Hospital, Tongji Medical College, Huazhong University of Science and Technology, Wuhan, China; 4 Department of Oral and Maxillofacial-Head and Neck Oncology, School and Hospital of Stomatology, Wuhan University, Wuhan, China

**Keywords:** Exosomes, Melanoma, Cancer-associated fibroblasts, Angiogenesis, Mmu-miR-155-5p, JAK2/STAT3 signaling pathway

## Abstract

**Background:**

Cancer-associated fibroblasts (CAFs) have been widely reported to promote tumor angiogenesis. However, the underlying mechanisms of the proangiogenic switch of CAFs remain poorly understood. This study aims to clarify the mechanisms underlying the proangiogenic switch of CAFs.

**Methods:**

NIH/3T3 cells were treated with B16 and B16F10-derived exosomes. Then the CAFs markers and proangiogenic factors were detected by RT-PCR and Western blot. CCK-8 assay, transwell migration assay, tube formation assay, and in vivo Matrigel plug assay were conducted to determine the proangiogenic capability of CAFs. Western blot and AG490 were used to investigate the role of Janus kinase 2/signal transducer and activator of transcription 3 (JAK2/STAT3) signaling pathway in the proangiogenic switch of CAFs. Bioinformatics analysis, luciferase reporter assay, microRNA mimic and inhibitor, and xenograft models were used to investigate the role of mmu-miR-155-5p (miR-155) in the proangiogenic switch of CAFs.

**Results:**

In this study, we show that melanoma cell-secreted exosomes can induce reprogramming of fibroblasts into CAFs and that exosomal miR-155 can trigger the proangiogenic switch of CAFs. Mechanistically exosomal miR-155 can be delivered into fibroblasts and promote the expression of proangiogenic factors, including vascular endothelial growth factor A (VEGFa), fibroblast growth factor 2 (FGF2), and matrix metalloproteinase 9 (MMP9), by directly targetin*g* suppressor of cytokine signaling 1 (SOCS1)*.* Downregulation of SOCS1 activates JAK2/STAT3 signaling pathway and elevates the expression levels of VEGFa, FGF2, and MMP9 in fibroblasts. Treatment with exosomes containing overexpressed miR-155 can promote angiogenesis, and the reduction of miR-155 in melanoma cell-secreted exosomes alleviates angiogenesis in vitro and in vivo.

**Conclusions:**

These results demonstrate that by promoting the expression of proangiogenic factors in recipient fibroblasts via SOCS1/JAK2/STAT3 signaling pathway, melanoma cell-secreted exosomal miR-155 can induce the proangiogenic switch of CAFs. Although tumor angiogenesis is modulated by various factors, exosomal miR-155 may be a potential target for controlling melanoma angiogenesis and used to set up novel strategies to treat melanoma.

**Electronic supplementary material:**

The online version of this article (10.1186/s13046-018-0911-3) contains supplementary material, which is available to authorized users.

## Background

Melanoma is a highly vascularized tumor. As several anti-angiogenic drugs have been approved to treat malignant tumors, the utility of anti-angiogenic strategies in treating melanoma has been confirmed [1]. However, recent studies and clinical trials have demonstrated the complexity of drug resistance to anti-angiogenic therapies in treatment of melanoma [2], driving the pressing demand for thorough investigation of the underlying mechanisms of melanoma angiogenesis.

Cancer-associated fibroblasts (CAFs), the activated form of tissue-resident fibroblasts, can promote tumor angiogenesis by secreting several proangiogenic cytokines, such as vascular endothelial growth factor A (VEGFa), fibroblast growth factor 2 (FGF2) and proteolytic enzymes, such as matrix metalloproteinases (MMPs) [3, 4]. However, the process of how tumor cells reprogram normal fibroblasts to proangiogenic CAFs remains incompletely understood.

Exosomes are small cell-released and lipid-bilayer-enclosed vesicles containing various bioactive proteins, mRNAs, and microRNAs (miRNAs). It serves as critical mediators in intercellular communication by transferring functional cargos to recipient cells [5]. Our previous study has shown that melanoma cell-secreted microvesicles can mediate the transformation of normal fibroblasts to CAFs and regulate the expression of vascular cell adhesion molecule-1, resulting in enhanced adhesion of melanoma cells and fibroblasts [6]. Tumor-released exosomal miRNAs have been shown to play a crucial role in reprogramming the tumor microenvironment [7]. Although various functions of tumor-secreted exosomal miRNAs have been well disclosed, the role of these miRNAs in the proangiogenic switch of CAFs remains poorly understood.

The Janus kinase 2/signal transducer and activator of transcription 3 (JAK2/STAT3) signaling pathway is activated in numerous types of tumors and regulates cell proliferation, angiogenesis, and migration of tumor cells. The activation of JAK2 protein triggers the phosphorylation of STAT3. The phosphorylated STAT3 dimerizes and translocates to the nucleus and then binds to targeted DNA elements and activates specific gene translation [8]. Studies have proved that the JAK2/STAT3 signaling pathway regulates the expression of proangiogenic factors, such as VEGFa and FGF2, and proteolytic enzymes, such as MMP9, and mediates numerous aspects of angiogenesis [9–11]. The suppressor of cytokine signaling (SOCS) proteins suppress JAK kinase capability and bind to the receptor to block STAT interaction. In particular, SOCS1 is a potent inhibitor of JAK2/STAT3 signaling cascade. The expression of SOCS1 reduces in various human cancers and is tightly associated with tumor angiogenesis [12, 13]. However, whether SOCS1 and JAK2/STAT3 pathway participate in the proangiogenic switch of CAFs and whether tumor-secreted exosomal miRNAs regulate both regulators are unclear.

In this study, we demonstrate that highly metastatic (B16F10) and weakly metastatic (B16) melanoma cell lines release and use exosomes to transfer mmu-miR-155-5p (miR-155) in fibroblasts. These exosomes induce CAF activation and elevate the expressions of proangiogenic factors (VEGFa, FGF2, and MMP9) in CAFs. Exosomal miR-155 directly targets SOCS1 and then activates the JAK2/STAT3 signaling pathway, leading to the proangiogenic switch of CAFs. These results may provide a novel therapeutic target for the anti-angiogenic therapy of melanoma treatment.

## Methods

### Reagents

Heat shock protein 90 (Hsp90) (Cat.No. 13171-1-AP), tumor susceptibility gene 101 (Tsg101) (Cat.No. 14497-1-AP), Calnexin (Cat.No. 10427-2-AP), CD63 (Cat.No. 25682-1-AP), MMP9 (Cat.No. 10375–2-AP), and VEGFa (Cat.No. 19003-1-AP) antibodies were purchased from Proteintech (Wuhan, China). Fibroblast activation protein (FAP) (Cat.No. ab53066) and α-smooth muscle actin (α-SMA) (Cat.No. ab124964) were obtained from Abcam. FGF2 antibody (Cat.No. sc-136255) was purchased from Santa Cruz. Phospho-JAK2 (P-JAK2) (Cat.No. 3771), JAK2 (Cat.No. 3230), phospho-STAT3 (P-STAT3) (Cat.No. 9145), STAT3 (Cat.No. 12640), SOCS1 (Cat.No. 3950), and Histone H3 (Cat.No. 4499) antibodies were purchased from Cell Signaling Technology. The growth factor-reduced Matrigel was acquired from BD Biosciences. Cell Counting Kit-8 (CCK-8) was purchased from Dojindo. Bicinchoninic acid (BCA) protein assay kits were obtained from Thermo Fisher.

### Cell lines and culture

NIH/3T3, B16, B16F10, A375 and MS-1 cells were purchased from the China Center for Type Culture Collection (Shanghai, China, 2016). The HGFs were isolated from the healthy gingival tissues of volunteers according to a previous study [14]. All procedures were in accordance with the Ethics Committee of School and Hospital of Stomatology, Wuhan University. STR was performed routinely on these cell lines to confirm their authenticity and Mycoplasma was routinely tested. NIH/3T3, B16F10, A375, HGF and MS-1 cells were cultivated in high-glucose Dulbecco’s Modified Eagle’s Medium (DMEM) (Hyclone, UT, USA) containing 10% fetal bovine serum (FBS) (Gibco, Carlsbad, CA, USA). B16 cells were cultivated in Roswell Park Memorial Institute (RPMI) 1640 medium (Hyclone, UT, USA) containing 10% FBS (Gibco, Carlsbad, CA, USA). All cell lines were cultivated at 37 °C in 5% CO_2_. When NIH/3T3 and A375 cells reached 70–80% confluence, melanoma-derived exosomes were added to the medium at 20 μg/mL.

### Isolation and analysis of exosomes

For exosome isolation, A375, B16 and B16F10 cells at 80% confluence were washed thrice with phosphate buffer solution (PBS) and then cultivated with growth medium containing 10% extracellular vesicles (EVs)-depleted FBS (prepared by overnight ultracentrifugation of medium-diluted FBS at 100,000 g at 4 °C). After 48 h, the conditioned medium (CM) was collected and pre-cleared by centrifugation at 800 g for 15 min and then at 10,000 g for 30 min. Exosomes were isolated by ultracentrifugation at 110,000 g for 70 min and washed in PBS by using the same ultracentrifugation conditions. Ultracentrifugation experiments were conducted with Beckman Optima L-100XP (Beckman Coulter, USA). Exosomes were observed by transmission electron microscopy HT7700 (HITACHI, Japan). The hydrodynamic diameter of exosomes was measured by using Nano-ZS ZEN 3600 (Malvern Instruments, UK).

### Exosome tracing

To monitor the interaction between exosomes and fibroblasts, the exosomes were labeled with PKH26 (Sigma-Aldrich, St. Louis, MO). After incubation with PKH26-labeled exosomes for 4 h, NIH/3T3 and HGF cells were observed by using a confocal microscope (Olympus FV1200, Japan).

### RNA extraction, RT-PCR and quantitative real-time PCR (qPCR)

Total RNA was extracted from cells and exosomes by using the TRIzol reagent (Takara, Tokyo, Japan), as instructed by the manufacturer. To analyze the expression levels of protein coding genes, RNA was reversely transcribed into cDNA by using PrimeScript RT Reagent Kit (Takara, Tokyo, Japan), followed by qPCR using SYBR® Premix Ex Taq™ II (Takara, Tokyo, Japan). MiRNA primers were synthesized by Sangon Biotech (Shanghai, China). For the quantification of mature miRNAs and U6 by qPCR, RNA was reverse-transcribed by using miRNA first-strand cDNA synthesis (Sangon Biotech, Shanghai, China), and then quantified by using MicroRNA qPCR Kit (SYBR Green Method) (Sangon Biotech, Shanghai, China). All processes were performed by following the manufacturer’s instructions. All RT-PCR tests were performed in triplicate. The comparative cycle threshold (Ct) method was used to quantify miRNA or mRNA levels by using U6 or glyceraldehyde 3-phosphate dehydrogenase (GAPDH) as the normalization control. QPCR was conducted on a QuantStudio™ 6 Flex (Life Technologies, USA). The Ct values should not differ by more than 0.5 among the triplicates. The sequences of the primers and the synthesized oligonucleotides used were listed (Additional file 1: Table S1).

### Protein extraction and Western blot analysis

Nuclear/cytoplasmic fractionation was separated by using Cell Fractionation Kit (Cell Signaling Technology, USA) according to the manufacturer’s instructions. The total protein of cells and exosomes was extracted by using M-PER (Pierce Inc., USA) supplemented with protease and phosphatase inhibitors on ice. The protein concentration of every sample was measured by using BCA Protein Assay Kit (Thermo Fisher Scientific Inc., USA). The mixture of the loading buffer (5×) and protein solutions was heated for 10 min at 95 °C. Aliquots of 20 μg of protein were added to 10% sodium dodecyl sulfate-polyacrylamide gel electrophoresis for 30 min at 60 V and for 1 h at 110 V. Afterward, the proteins were transferred to polyvinylidene difluoride (PVDF) membrane in transfer buffer for 2 h at 200 mA. The membranes were blocked with 5% skim milk in Tris-buffered saline containing 0.05% Tween 20 (TBST) at room temperature for 1 h. When phosphorylated protein was detected, the PVDF membrane was blocked with TBST with 5% BSA. Then, the membranes were incubated with antibodies overnight at 4 °C. Subsequently, the bound antibodies were detected by horseradish peroxidase-conjugated, anti-mouse IgG or anti-rabbit IgG (Pierce Chemical, Rockford, IL, USA). Western blot analyses were repeated thrice to confirm the results.

### Enzyme-linked immunosorbent assay (ELISA)

The concentrations of VEGFa, FGF2, and MMP9 in the culture medium of NIH/3T3 cells were measured by ELISA kits (4A Biotech Co., Ltd., China) following the manufacturer’s instructions and analyzed by comparing the optical densities of the samples with the standard curve of the kits.

### Cell proliferation assay

MS-1 cells were seeded in 96-well culture plates and cultivated by the CM of fibroblasts, which were stimulated with or without melanoma-secreted exosomes. After incubation for 24, 48, and 72 h, cell proliferation was determined by CCK-8 (Dojindo, Japan) in accordance with the manufacturer’s instructions.

### Migration and tube formation assays

For the migration assay, 5 × 10^4^ MS-1 cells were seeded in 100 μl of serum-free medium in the upper chambers of 24-well plates with inserts (Corning, USA). Fibroblasts treated with or without melanoma-secreted exosomes were seeded in 600 μl of 10% FBS-DMEM in the lower chambers. After 24 h incubation, the cells in the upper chamber were removed, and cells that traversed to the reverse face were fixed and stained with crystal violet in accordance with the manufacturer’s instructions. Six random fields were counted. Migration assays were performed in triplicate. For tube formation assay, the MS-1 cells (1 × 10^4^/well) were seeded in 48-well plates precoated with Matrigel (BD Biosciences, San Jose, CA, USA) and cultivated in the CM of fibroblasts stimulated with or without melanoma-secreted exosomes. After 6 h incubation, the capillary-like structure was observed and photographed under a microscope (BHS-313 Olympus). Quantification and analysis of tube formation results were performed using Image-Pro Plus 6.0 (Media Cybernetics, Inc., USA).

### In vivo angiogenesis study

Fibroblasts (5 × 10^6^) stimulated with B16 and B16F10-derived exosomes and the untreated fibroblasts were mixed with Matrigel (200 μL) and subcutaneously injected into C57BL/6 mice (male, 8-week-old) (*n* = 5). In each mouse, three types of fibroblast-Matrigel mixture were injected into the armpits of the left upper, right upper, and right lower limbs. All mice were raised in sterile laminar flow cabinets under appropriate pathogen-free conditions with a 12 h–12 h light-dark cycle. After 1 week, the Matrigel plugs were harvested and processed for analysis. All mice were raised and manipulated following the protocols approved by the Laboratory Animal Care and Use Committee of Wuhan University (approval numbers: S07917110A).

B16 (4 × 10^6^) and B16F10 (4 × 10^6^) cells and different types of exosome-treated NIH/3T3 cells were harvested from culture plates, premixed with Matrigel (200 μL) at the ratio of 1:1, and subcutaneously inoculated into the armpits of the left upper, right upper, and right lower limbs of C57BL/6 mice (male, 8-week-old) (Fig. 7a and g). After 1 week, xenografts were harvested. The xenografts were weighed and measured by using a caliper, measured in accordance with the formula: volume (cm^3^) = (width^2^ × length)/2, and then embedded in paraffin for immunofluorescence staining.

### Immunofluorescence

The Matrigel plugs and xenografts were extracted and then fixed in 4% paraformaldehyde for 24 h, dehydrated using graded ethanol, embedded in paraffin, sectioned serially, and incubated with anti-mouse CD31 antibody (GB11063–3, Servicebio, Wuhan), and then with Cy3-conjugated secondary IgG (Servicebio, Wuhan). Analyses were performed with a fluorescent microscope (Biozero BZ-8000, Keyence, Osaka, Japan). Microvessel density (MVD) was quantified from six random microscopic fields. Any single cell or discrete cluster stained for CD31 was counted as one microvessel as reported previously [15].

After incubation with B16- and B16F10-secreted exosomes for 6 h, NIH/3T3 cells were fixed with 4% paraformaldehyde for 10 min, followed by incubation with 0.2% Triton X-100, and blocked with FBS for 30 min at room temperature. The slides were incubated with anti-P-STAT3 antibody overnight at 4 °C, followed by incubation with FITC-conjugated goat anti-rabbit IgG antibody for 45 min at room temperature. Nuclear staining was then incubated with DAPI. The stained cells were examined and photographed using a confocal microscope (Olympus FV1200, Japan).

### RNA oligoribonucleotides

FITC-labeled miR-103, miR-155 mimic and miR-155 inhibitor (anti-miR-155), and their negative control (miR-NC and anti-NC, respectively) were purchased from Sangon (Shanghai, China).

### Luciferase reporter assay

Luciferase plasmids (300 ng) (pGL3) encoding wild-type or mutant 3′ untranslated region (3′UTR) of SOCS1 were co-transfected with miR-155 mimic or miR-NC and anti-miR-155 or anti-NC. Lipo6000™ (Beyotime, China) was used as the transfectant. At 48 h after transfection into 293 T cells, luciferase activity was measured by using a Dual-Luciferase Reporter Assay Kit (Promega, USA) in accordance with the manufacturer’s instructions.

### Statistical analysis

Results are expressed as means ± standard error of the mean (SEM) from triplicates of independent experiments and analyzed by student’s t-tests or one-way ANOVA by using the Statistical Product and Service Solutions (SPSS) 21 software (SPSS Inc., Chicago, USA). Results were considered statistically significant when *P* < 0.05.

## Results

### Identification of exosomes secreted by melanoma cells

**Fig. 1:**
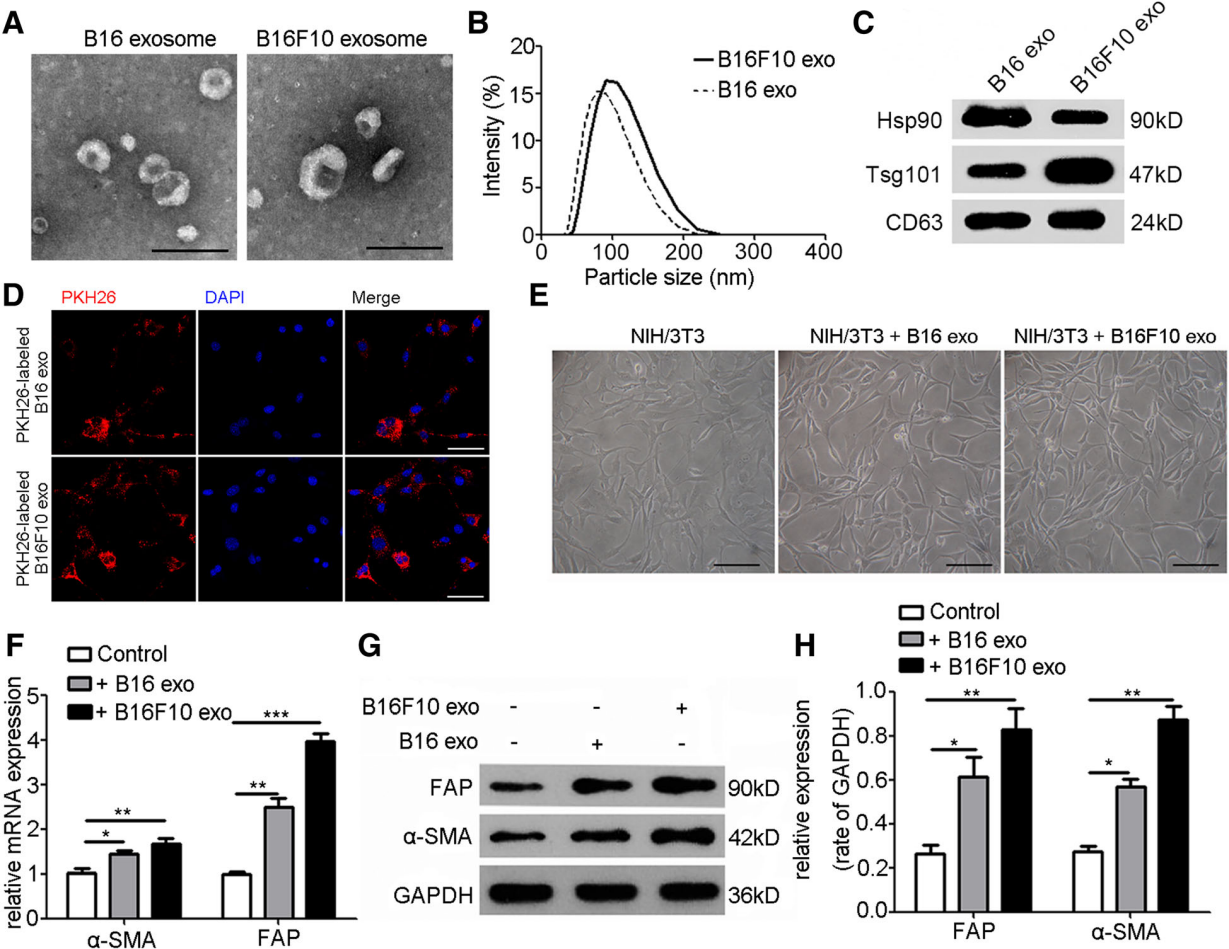
Melanoma cell-secreted exosomes transform fibroblasts into CAFs. **a**, **b** Exosomes released by B16 and B16F10 cells were identified by using transmission electron microscope and dynamic light scattering analysis. Scale bar, 200 nm. **c** Characterization of exosomes with Western blot analysis. Exosomal markers: Hsp90, Tsg101, and CD63. **d** Confocal microscope images showed the uptake of PKH26-labeled exosomes in NIH/3T3 cells. Scale bar, 50 μm. **e** The phase morphology of NIH/3T3 cells with different treatments. Scale bar, 50 μm. **f** The expressions of α-SMA and FAP were investigated by RT-PCR after NIH/3T3 cells were treated with exosomes (20 μg/mL) extracted from B16 and B16F10 cells for 24 h. GAPDH was used as the normalization control. **g**, **h** The expressions of α-SMA and FAP in NIH/3T3 cells with different treatments were evaluated by Western blot and densitometry analysis. Independent experiments performed in triplicate. * *P* < 0.05, ** *P* < 0.01, *** *P* < 0.001 vs. control group. Values are represented as means ± SEM. CAFs: cancer-associated fibroblasts. DAPI: 4′6-diamidino-2-phenylindole. Exo: exosomes. FAP: fibroblast activation protein. GAPDH: Glyceraldehyde 3-phosphate dehydrogenase. Hsp90: heat shock protein 90. SEM: standard error of the mean. Tsg101: tumor susceptibility gene 101. α-SMA: α-smooth muscle actin

Isolated from the culture supernatant of B16 and B16F10 cells, the EVs were identified by using transmission electron microscope and dynamic light scattering analysis (Fig. 1a, b). Western blot was performed to detect the exosomal markers Hsp90, Tsg101, and CD63 (Fig. 1c). Bilayer-enclosed morphology, diameters of 50–200 nm, and the existence of exosomal markers verified that the vesicles were exosomes.

### Melanoma cell-secreted exosomes reprogram fibroblasts into proangiogenic CAFs

**Fig. 2:**
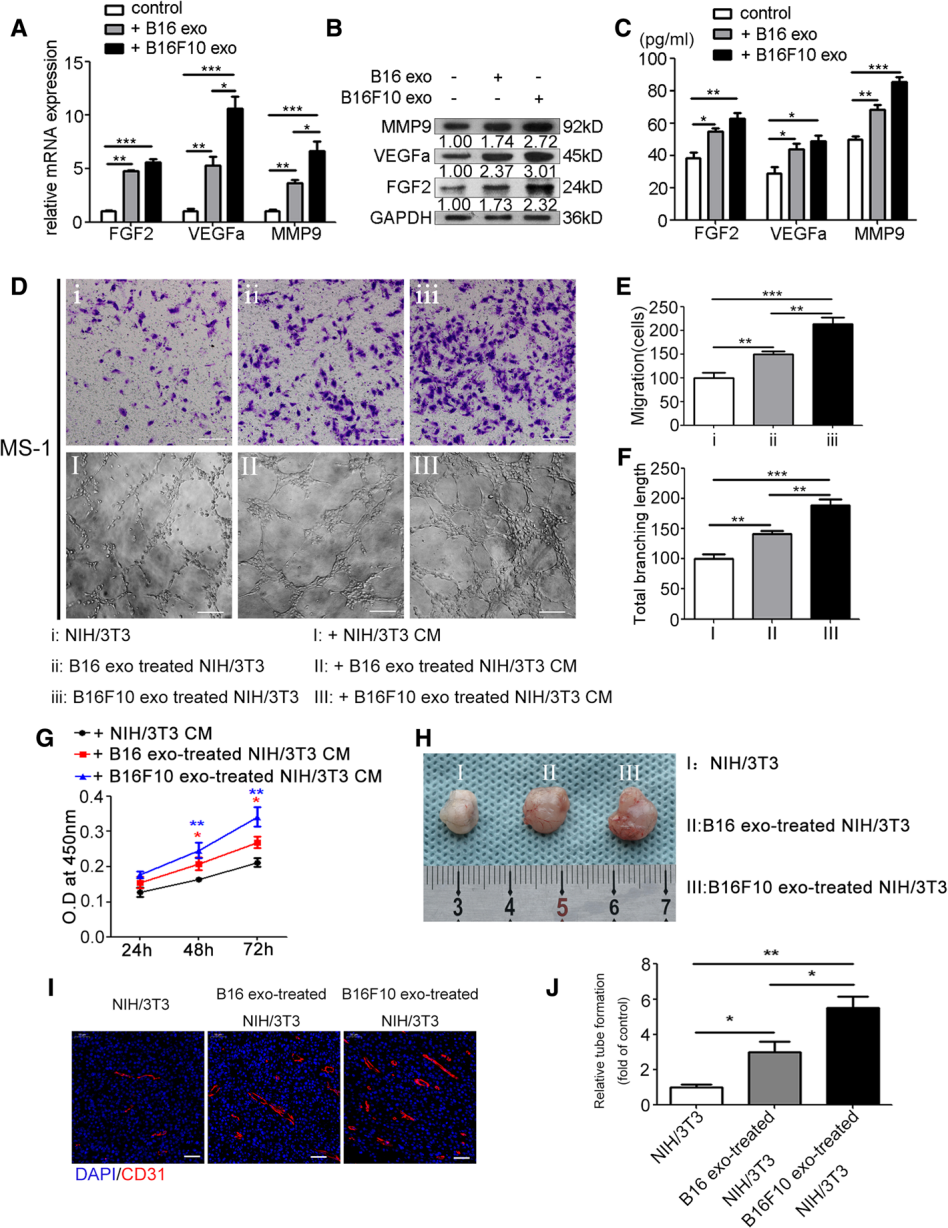
Melanoma cell-secreted exosomes reprogram fibroblasts into proangiogenic CAFs in vitro and in vivo. Treatment with B16- and B16F10-secreted exosomes for 24 h significantly upregulated the expressions of FGF2, VEGFa, and MMP9 in NIH/3T3 cells as shown by RT-PCR (**a**) and Western blot (**b**) (The ratio of the expressions of MMP9, VEGFa, and FGF2 is indicated below the panels), and promoted the secretion of these proangiogenic factors as shown by ELISA (**c**). Independent experiments performed in triplicate. * *P* < 0.05, ** *P* < 0.01, *** *P* < 0.001 vs. control group. **d**, **e**, **f**, and **g** CCK-8 assay, transwell migration assay, and tube formation assay showed that the CM from NIH/3T3 cells treated with B16- and B16F10-secreted exosomes promoted MS-1 cell proliferation, migration, and tube formation. Independent experiments performed in triplicate. * *P* < 0.05, ** *P* < 0.01, *** *P* < 0.001 vs. control group. Scale bar, 50 μm. One-way ANOVA and student’s t-tests. **h** Representative photographs of Matrigel plugs harvested at 1 week after subcutaneous injection into C57BL/6 mice (*n* = 5). **i** Representative immunofluorescence images of the neovessels in the Matrigel plugs. Vasculature and nuclear staining were performed by using CD31 (red) and DAPI (blue), respectively. Scale bar, 50 μm. **j** Quantitative analysis of the neovessels in the Matrigel plugs**.** * *P* < 0.05, ** *P* < 0.01, student’s t-tests. Values are represented as means ± SEM. CAFs: cancer-associated fibroblasts. CCK-8: Cell Counting Kit-8. CM: conditioned medium. DAPI: 4′6-diamidino-2-phenylindole. ELISA: Enzyme-linked immunosorbent assay. Exo: exosomes. FGF2: fibroblast growth factor 2. GAPDH: Glyceraldehyde 3-phosphate dehydrogenase. MMP9: matrix metalloproteinase 9. SEM: standard error of the mean. VEGFa: vascular endothelial growth factor A

To explore whether melanoma-secreted exosomes can be absorbed by fibroblasts, exosomes were fluorescently labeled with PKH26 and then cocultivated with NIH/3T3 cells. After 6 h incubation, the cytoplasm of NIH/3T3 cells was fluorescently stained (Fig. 1d). Stimulated with B16- and B16F10-secreted exosomes for 24 h, NIH/3T3 cells featured a spindle-like shape, which was regarded as the typical morphology of CAFs (Fig. 1e). Western blot and RT-PCR showed that treatment with B16- and B16F10-secreted exosomes significantly upregulated the expression of CAF typical markers, e.g., α-SMA and FAP (Fig. 1f, h). Meanwhile, exosomes derived from human melanoma cell line A375 could be absorbed by human gingival fibroblasts (HGFs) (Additional file 1: Figure S1A, 1B, 1C and 1D). Interestingly, treatment with A375-secreted exosomes significantly upregulated the expression of α-SMA and FAP in HGFs, and transformed HGFs into spindle-like shape as well (Additional file 1: Figure S1E, 1F, 1G and 1H). CAFs have been reported to promote tumor angiogenesis by secretion of proangiogenic factors. NIH/3T3 cells were treated with exosomes (20 μg/mL) secreted by B16 and B16F10 cells. After 24 h stimulation, RT-PCR and Western blot showed that the expressions of typical proangiogenic factors MMP9, VEGFa, and FGF2 remarkably increased (Fig. 2a, b; Additional file 1: Figure S2). ELISA showed that the concentrations of MMP9, VEGFa, and FGF2 in the culture supernatant of NIH/3T3 cells treated with B16- and B16F10-secreted exosomes were significantly higher than those of the untreated group (Fig. 2c).

We explored whether fibroblasts treated by melanoma cell-secreted exosomes can promote angiogenesis in vitro. The endothelial cell (EC) line MS-1 was used. CCK-8 assay showed that the CM from NIH/3T3 cells treated with B16- and B16F10-secreted exosomes remarkably promoted MS-1 cell proliferation (Fig. 2g). Transwell migration assay indicated that the number of traversed MS-1 cells significantly increased when incubated with the CM from NIH/3T3 cells treated with B16- and B16F10-secreted exosomes (Fig. 2d, e). In the Matrigel tube formation assay, the total branching length of the tubes formed by MS-1 significantly increased when incubated with the CM from NIH/3T3 cells treated with B16- and B16F10-secreted exosomes (Fig. 2d and f).

To investigate the proangiogenic capability of fibroblasts treated with melanoma cell-secreted exosomes in vivo, NIH/3T3 cells treated with B16- and B16F10-secreted exosomes and the nontreated NIH/3T3 cells were injected subcutaneously into C57BL/6 mice. After 1 week, the Matrigel plugs were harvested and processed for Immunofluorescence staining. Then the microvessel density (MVD) was quantified. In comparison with the control group, the MVDs of B16- and B16F10-secreted exosome-treated groups increased significantly (Fig. 2h, i and j).

### Melanoma cell-secreted exosomes suppress the expression of SOCS1 and activate the JAK2/STAT3 signaling pathway, which regulates the proangiogenic switch of CAFs

**Fig. 3:**
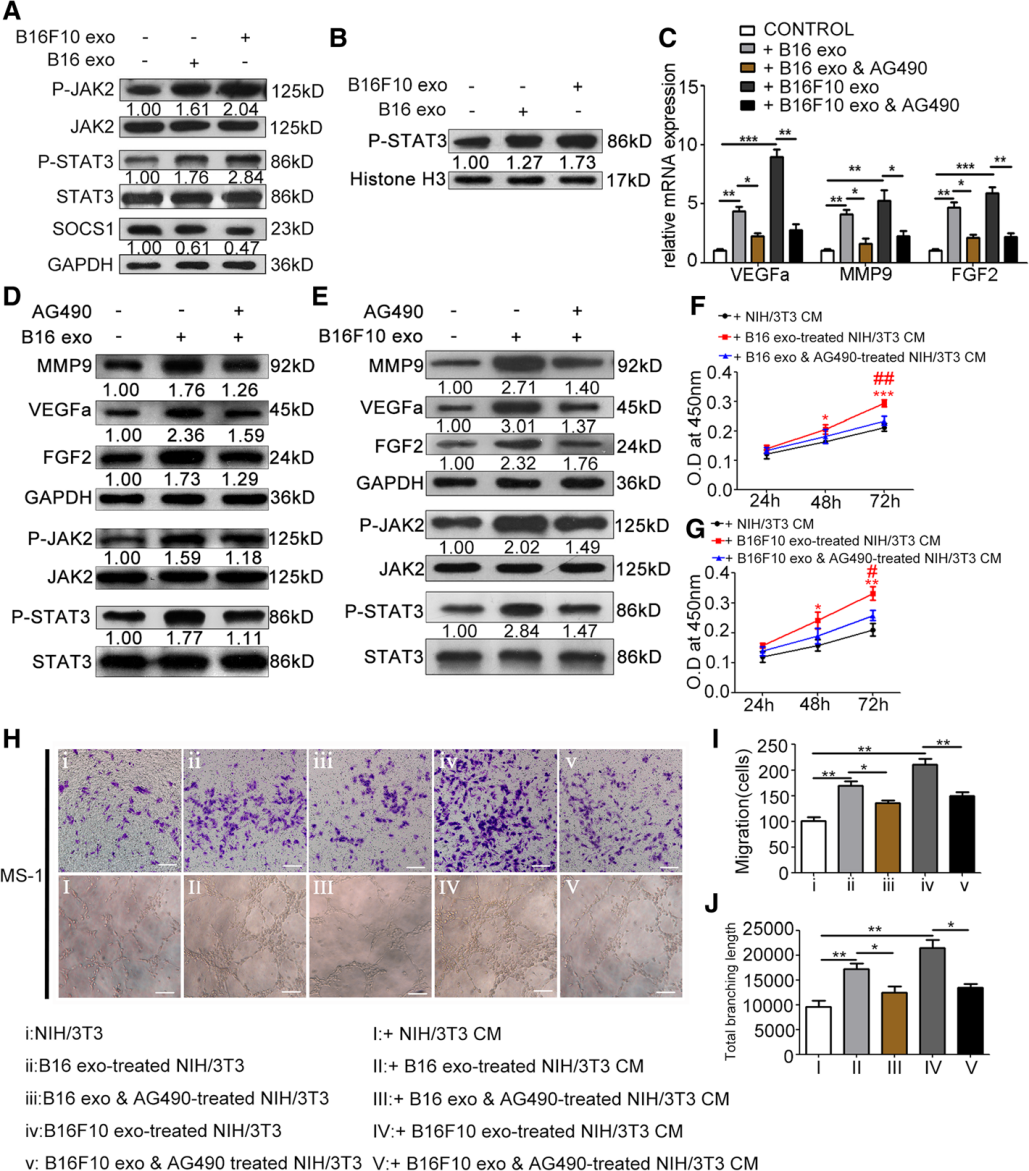
Melanoma cell-secreted exosomes regulate the proangiogenic switch of CAFs via SOCS1/JAK2/STAT3 signaling pathway. **a** Treatment with B16- and B16F10-secreted exosomes elevated the phosphorylation levels of JAK2 and STAT3 and suppressed the expression of SOCS1. The ratio of the expression of SOCS1 and the phosphorylation levels of JAK2 and STAT3 are indicated below the panels. **b** Western blotting of nuclear P-STAT3 expression. The nuclear protein Histone H3 was used as the nuclear protein marker. **c** Treatment with AG490 downregulated the mRNA expressions of VEGFa, MMP9, and FGF2 in CAFs. * *P* < 0.05, ** *P* < 0.01, *** *P* < 0.001. One-way ANOVA and student’s t-tests. **d**, **e** Treatment with AG490 downregulated the phosphorylation levels of JAK2 and STAT3, and the expressions of VEGFa, MMP9, and FGF2 in CAFs. **f**, **g** The promotive effect of the CM from NIH/3T3 cells treated with B16- and B16F10-secreted exosomes on MS-1 cell proliferation was blocked by AG490. * *P* < 0.05, ** *P* < 0.01, *** *P* < 0.001 vs. the CM from NIH/3T3 cells; # *P* < 0.05, ## *P* < 0.01 vs. the CM from NIH/3T3 cells treated with B16- and B16F10-secreted exosomes. **h**, **i**, and **j** The promotive effects of the CM from NIH/3T3 cells treated with B16- and B16F10-secreted exosomes on MS-1 cell migration and tube formation were blocked by AG490. * *P* < 0.05, ** *P* < 0.01. Independent experiments performed in triplicate. Values are expressed as means ± SEM. One-way ANOVA and student’s t-tests. Scale bar, 50 μm. CAFs: cancer-associated fibroblasts. CM: conditioned medium. Exo: exosomes. FGF2: fibroblast growth factor 2. GAPDH: Glyceraldehyde 3-phosphate dehydrogenase. JAK2: Janus kinase 2. MMP9: matrix metalloproteinase 9. SEM: standard error of the mean. SOCS1: suppressor of cytokine signaling 1. STAT3: signal transducer and activator of transcription 3. VEGFa: vascular endothelial growth factor A

After treatment of exosomes extracted from B16 and B16F10 cells, Western blot analysis revealed the significantly elevated phosphorylation levels of JAK2 and STAT3, and increased nuclear accumulation of P-STAT3 in NIH/3T3 cells (Fig. 3a, b). Besides, confocal microscope verified the enhanced nuclear accumulation of P-STAT3 in NIH/3T3 cells after treatment of B16- and B16F10-secreted exosomes (Additional file 1: Figure S3). To investigate whether the activation of JAK2/STAT3 signaling pathway resulted in upregulation of MMP9, VEGFa, and FGF2, JAK2 inhibitor AG490 was used to concurrently treat NIH/3T3 cells with melanoma cell-secreted exosomes. RT-PCR and Western blot showed that treatment with AG490 alleviated the promoting effect of B16- and B16F10-secreted exosomes on the phosphorylation of JAK2 and STAT3, and expressions of MMP9, VEGFa, and FGF2 (Fig. 3c, d and e). Treatment with AG490 also weakened the promoting effect of the CM of NIH/3T3 cells treated with B16- and B16F10-secreted exosomes on MS-1 cell proliferation (Fig. 3f, g), migration (Fig. 3h, i), and tube formation (Fig. 3h and j). As an endocellular suppressor of the JAK/STAT signaling pathway, SOCS1 was significantly downregulated in NIH/3T3 cells after treatment of B16- and B16F10-secreted exosomes (Fig. 3a; Additional file 1: Figure S4).

### Delivery of melanoma cell-secreted exosomal miR-155 into CAFs

**Fig. 4:**
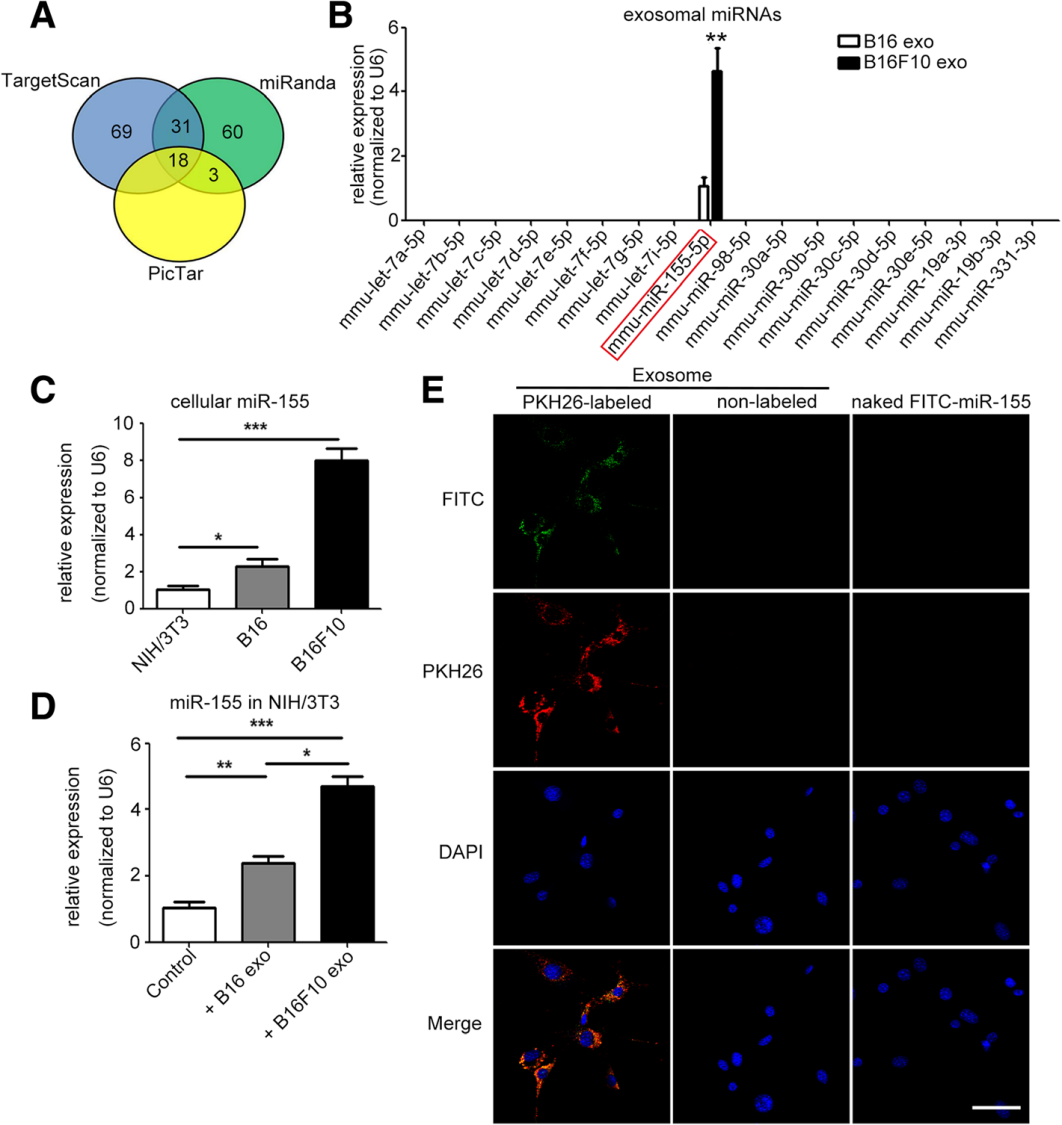
Delivery of melanoma cell-secreted exosomal miR-155 into fibroblasts. **a** MiRNAs that might target SOCS1 were predicted with TargetScan, miRanda, and PicTar. **b** The predicted candidate miRNAs in B16- and B16F10-secreted exosomes were investigated by qRT-PCR. ** *P* < 0.01 vs. B16-secreted exosomes. Student’s t-tests. Independent experiments performed in triplicate. Values are expressed as means ± SEM. **c** Comparison of the miR-155 levels in NIH/3T3, B16, and B16F10 cells. **d** The levels of miR-155 in recipient NIH/3T3 cells after treatment with B16- and B16F10-secreted exosomes. * *P* < 0.05, ** *P* < 0.01, *** *P* < 0.001. Independent experiments performed in triplicate. Values are expressed as means ± SEM. One-way ANOVA and student’s t-tests. **e** Exosomes from B16F10 cells that were transfected with 40 nM FITC-tagged miR-155 were labeled with fluorescent dye PKH26 (left panel) and then applied to treat NIH/3T3 cells for 4 h, following by imaging under a fluorescence microscope. As negative controls, NIH/3T3 cells were incubated with non-labeled exosomes (exosomes were treated with PBS instead of PKH26, middle panel) or 40 nM naked FITC-tagged miR-155 (FITC-tagged miR-155 was added directly to the NIH/3T3 cells, right panel) for 4 h. FITC: FITC-tagged miR-155 (green), PKH26: PKH26-labeled exosomes (red), DAPI: cell nuclei (blue). Scale bar, 50 μm. DAPI: 4′6-diamidino-2-phenylindole. Exo: exosomes. FITC: fluorescein isothiocyanate. MiR-155: mmu-miR-155-5p. MiRNAs: microRNAs. SEM: standard error of the mean. SOCS1: suppressor of cytokine signaling 1

To predict the miRNAs that target the SOCS1 gene, we applied bioinformatics analysis based on TargetScan, miRanda, and PicTar (Fig. 4a). The candidate miRNAs overlapping in the three databases were detected by qPCR. Among these miRNAs, miR-155 was the most abundant in B16- and B16F10-secreted exosomes (Fig. 4b). Both exosomal miR-155 and cellular miR-155 were significantly differentially expressed. Cellular miR-155 and exosomal miR-155 were detected at relatively high levels in B16F10 cells, at medium levels in B16 cells, and at low levels in NIH/3T3 cells (Fig. 4b, c). Furthermore, cellular miR-155 increased intensely in recipient NIH/3T3 cells after treatment with B16F10-secreted exosomes. Cellular miR-155 increased mildly in NIH/3T3 cells after treatment with B16-secreted exosomes (Fig. 4d). After transfection with miR-155 mimic, miR-155 increased in B16 cells and B16-secreted exosomes (Additional file 1: Figure S5A-B). Furthermore, miR-155 levels in recipient NIH/3T3 cells remarkably increased after treatment with exosomes from miR-155-mimic-transfected B16 cells (Additional file 1: Figure S5C). After transfection with anti-miR-155, miR-155 decreased in B16F10 cells and B16F10-secreted exosomes (Additional file 1: Figure S5D-E). Furthermore, miR-155 levels in recipient NIH/3T3 cells remarkably increased after treatment with exosomes from anti-NC-transfected B16F10 cells, but showed no change when treated with exosomes from anti-miR-155-transfected B16F10 cells (Additional file 1: Figure S5F). Exosomes from B16F10 cells transfected with fluorescein isothiocyanate (FITC)-tagged miR-155 were further labeled with fluorescent dye PKH26 and then used to incubate with NIH/3T3 cells. Both FITC and PKH26 fluorescence were observed within recipient NIH/3T3 cells. However, no FITC or PKH26 fluorescence was observed in recipient NIH/3T3 cells treated with non-labeled exosomes or naked FITC-tagged miR-155 (Fig. 4e). These results indicate that melanoma cell-secreted exosomal miR-155 can be delivered into CAFs.

### MiR-155 targets 3′UTR of SOCS1

**Fig. 5:**
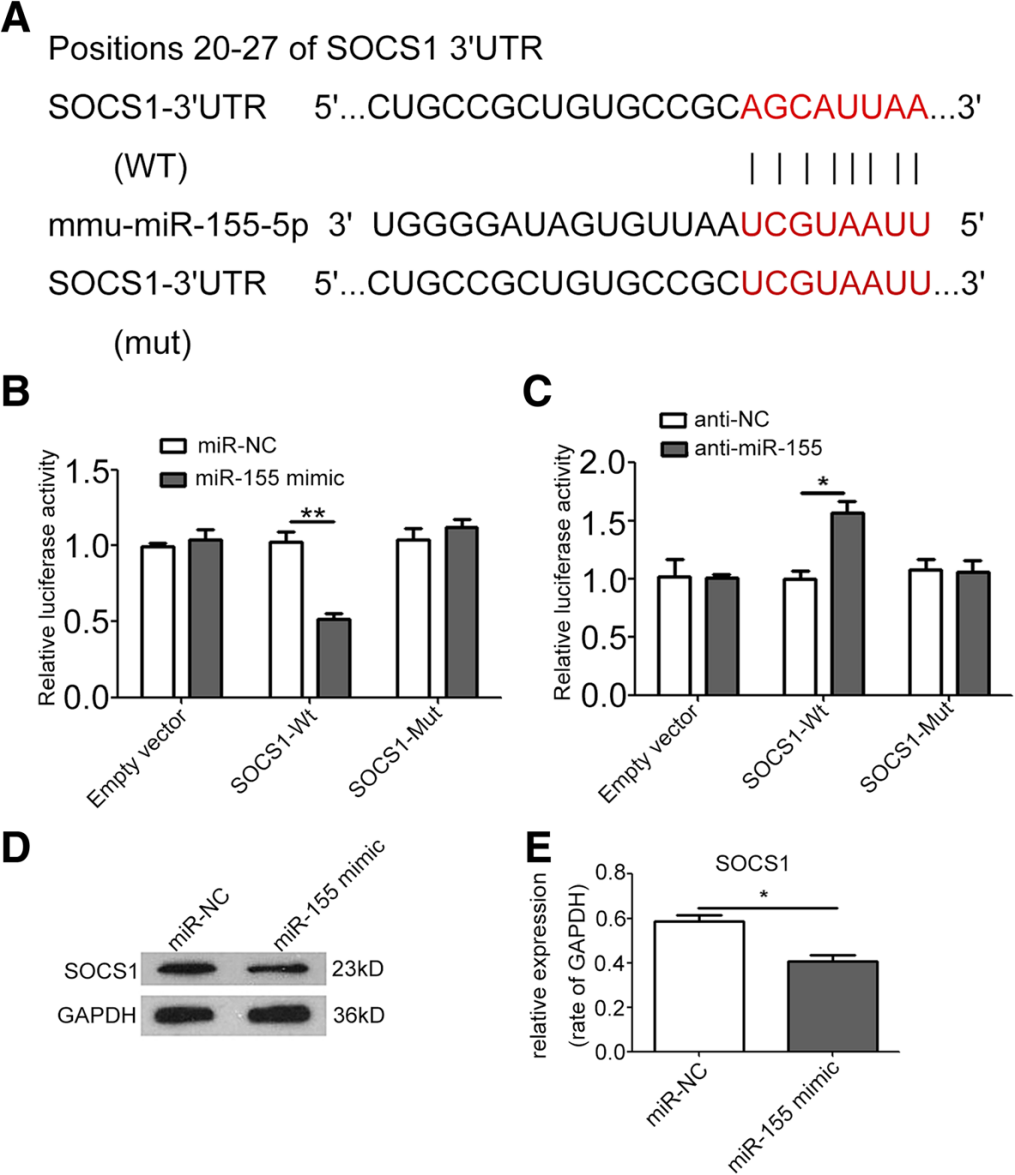
MiR-155 targets 3′UTR of SOCS1. **a** The wild-type and a mutated type of binding site between miR-155 and SOCS1. **b**, **c** Overexpression of miR-155 reduced SOCS1–3′UTR luciferase activity in vitro but not mutated SOCS1–3′UTR luciferase activity. Inhibition of miR-155 resulted in the opposite effect. **d**, **e** Western blot and densitometry analysis showed that overexpression of miR-155 suppressed the expression of SOCS1 in NIH/3T3 cells. * *P* < 0.05, ** *P* < 0.01. Student’s t-tests. Independent experiments performed in triplicate. Values are expressed as means ± SEM. Anti-NC: inhibitor negative control. Anti-miR-155: miR-155 inhibitor. GAPDH: Glyceraldehyde 3-phosphate dehydrogenase. MiR-NC: miR-negative control. Mut: mutated type. SEM: standard error of the mean. SOCS1: suppressor of cytokine signaling 1. WT: wild type. 3′UTR: 3′ untranslated region

After detailed sequence analysis, we observed that 3**′**UTR of SOCS1 exhibited a putative binding site of miR-155 at positions 20–27 (Fig. 5a). Dual-luciferase reporter assay revealed that co-transfection of miR-155 mimic significantly inhibited the activity of firefly luciferase reporter carrying the wild-type 3′UTR of SOCS1 (Fig. 5b), whereas co-transfection of anti-miR-155 significantly increased the activity of firefly luciferase reporter carrying the wild-type 3′UTR of SOCS1 (Fig. 5c). Furthermore, treatment with miR-155 mimic significantly suppressed the expression of SOCS1 in NIH/3T3 cells (Fig. 5d, e).

### Exosomal miR-155 regulates the proangiogenic phenotype of CAFs in vitro and in vivo

**Fig. 6:**
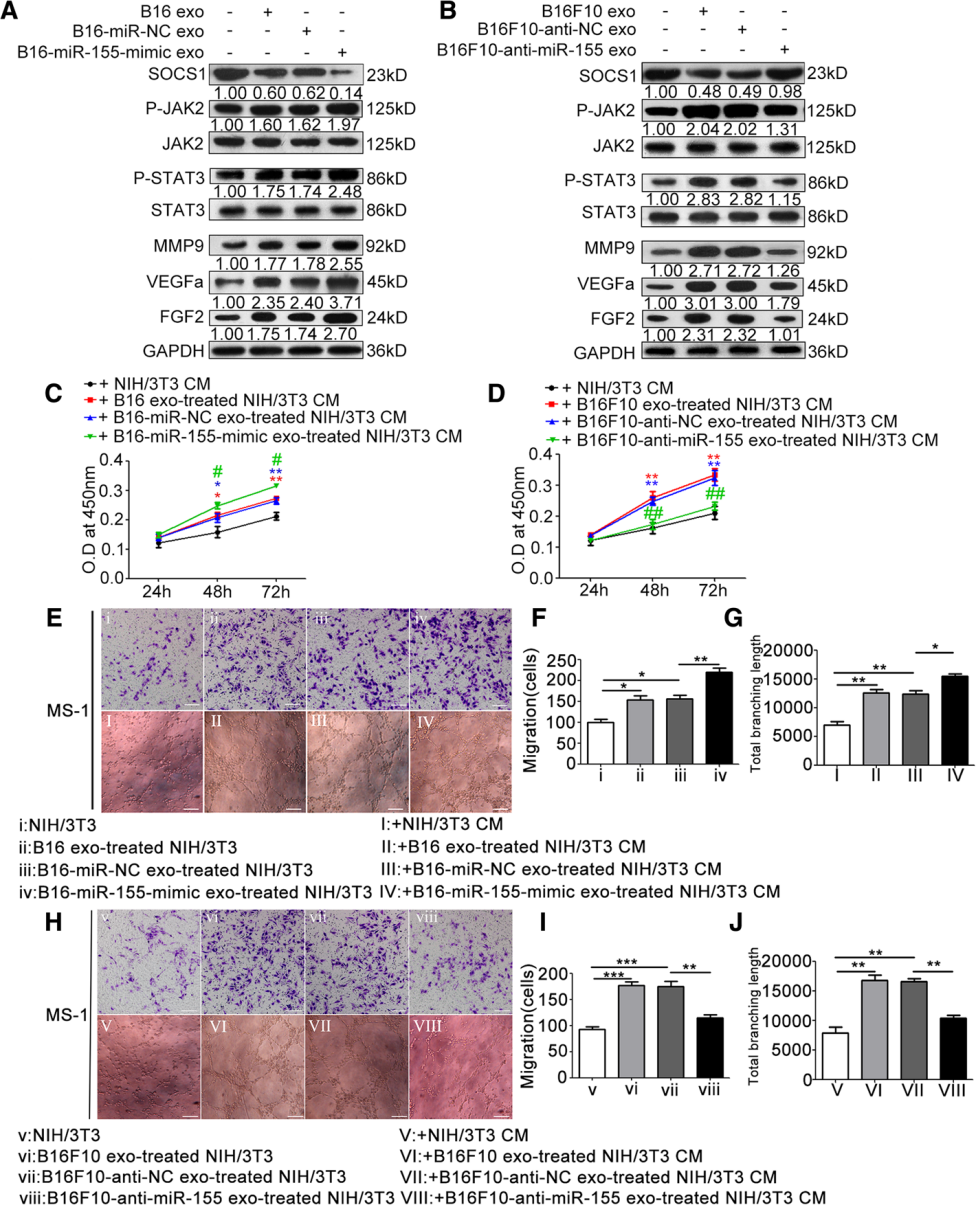
Exosomal miR-155 regulates the proangiogenic switch of CAFs in vitro. **a**, **b** Western blot and densitometry analysis showed that treatment with exosomes from miR-155-mimic-transfected B16 cells downregulated the expression of SOCS1, elevated the phosphorylation levels of JAK2 and STAT3, and upregulated the expressions of MMP9, VEGFa, and FGF2. Whereas treatment with exosomes from anti-miR-155-transfected B16F10 cells resulted in the opposite effects. The ratios of the expressions of SOCS1, MMP9, VEGFa, and FGF2, and the phosphorylation levels of JAK2 and STAT3 are indicated below the panels. **c**, **d** CCK-8 assay showed that the CM from NIH/3T3 cells treated with exosomes extracted from miR-155-mimic-transfected B16 cells promoted MS-1 cell proliferation. Whereas the CM from NIH/3T3 cells treated with exosomes extracted from anti-miR-155-transfected B16F10 cells had the opposite effect. * *P* < 0.05, ** *P* < 0.01 vs. the CM from nontreated NIH/3T3 cells. # *P* < 0.05, ## *P* < 0.01 vs. the miR-NC-transfected group or anti-NC-transfected group. Student’s t-tests. **e**, **f**, and **g)** Overexpression of miR-155 in exosomes enhanced the promotive effects of the CM from NIH/3T3 cells that treated with miR-155-mimic-transfected B16-secreted exosomes on MS-1 cell proliferation and tube formation. Whereas reduction of miR-155 in exosomes resulted in the opposite effects (**h**, **i**, and **j**). * *P* < 0.05, ** *P* < 0.01, *** *P* < 0.001. Independent experiments performed in triplicate. Values are expressed as means ± SEM. Student’s t-tests. Scale bar, 50 μm. Anti-NC: inhibitor negative control. Anti-miR-155: miR-155 inhibitor. CAFs: cancer-associated fibroblasts. CM: conditioned medium. Exo: exosomes. FGF2: fibroblast growth factor 2. GAPDH: Glyceraldehyde 3-phosphate dehydrogenase. JAK2: Janus kinase 2. MiR-NC: miR-negative control. MMP9: matrix metalloproteinase 9. SEM: standard error of the mean. SOCS1: suppressor of cytokine signaling 1. STAT3: signal transducer and activator of transcription 3. VEGFa: vascular endothelial growth factor A

**Fig. 7:**
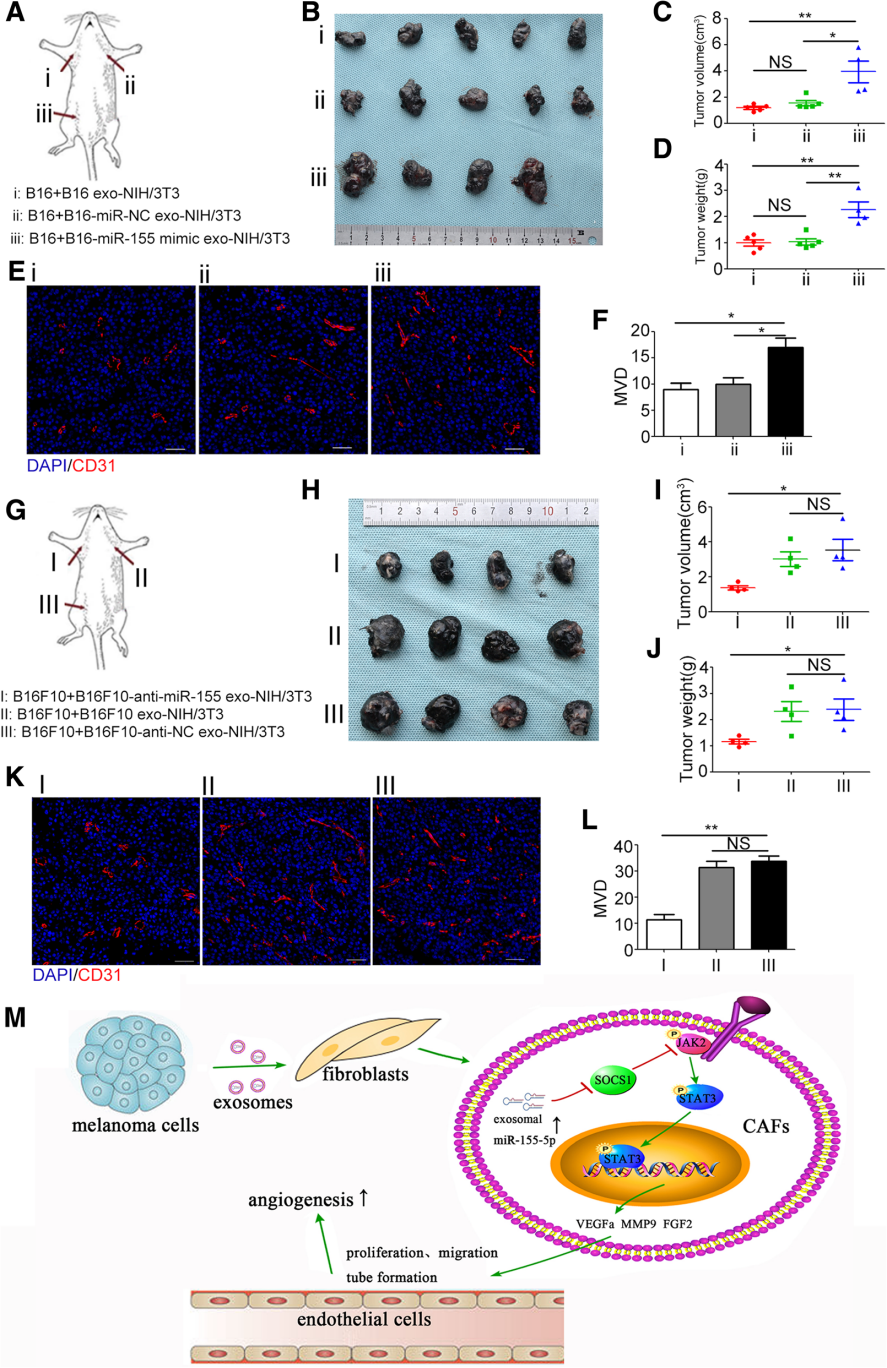
Exosomal miR-155 regulates angiogenesis in vivo. **a**, **g** The schematic drawing show the positions where the cells were injected. **b**, **h** The pictures show the isolated tumors. The tumors in the same positions in each group are from the same mouse. **c**, **d**, **i**, and **j** The tumor size and weight (means ± SEM) of different treatment groups. * *P* < 0.05, ** *P* < 0.01. **e**, **f**, **k**, and **l** Representative fluorescence microscopy images and the quantitative analysis of MVD of the xenografts. * *P* < 0.05. ** P < 0.01. Student’s t-tests. Scale bar, 50 μm. Anti-NC: inhibitor negative control. Anti-miR-155: miR-155 inhibitor. DAPI: 4′6-diamidino-2-phenylindole. Exo: exosomes. MiR-NC: miR-negative control. MVD: microvessel density

To investigate the function of exosomal miR-155 in CAFs, we treated NIH/3T3 cells with exosomes from miR-155-mimic-transfected B16 cells and anti-miR-155-transfected B16F10 cells. In miR-155-mimic-transfected B16-secreted exosome-treated group, SOCS1 expression was significantly downregulated, and the phosphorylation levels of JAK2 and STAT3 and expressions of MMP9, VEGFa, and FGF2 were significantly elevated (Fig. 6a). Conversely, treatment with exosomes from anti-miR-155-transfected B16F10 cells significantly upregulated the expression of SOCS1 but downregulated the phosphorylation levels of JAK2 and STAT3 and expressions of MMP9, VEGFa, and FGF2 (Fig. 6b). The CM of NIH/3T3 cells treated with exosomes from miR-155-mimic-transfected B16 cells showed enhanced effects on MS-1 cell proliferation (Fig. 6c), migration (Fig. 6e-f), and tube formation (Fig. 6e and g), whereas that from anti-miR-155-transfected B16F10 cells showed reduced effects on MS-1 cell proliferation (Fig. 6d), migration (Fig. 6h, i), and tube formation (Fig. 6h and j). The xenograft models showed consistent results with in vitro assays. Upregulation of miR-155 in B16-secreted exosomes significantly increased the MVD of xenografts (Fig. 7a, b, and e, f), whereas downregulation of miR-155 in exosomes resulted in the opposite effect (Fig. 7g, h, and k, l).

## Discussion

In melanoma, EVs, such as exosomes and microvesicles, have been reported to play a predominant role in promoting pre-metastatic niche formation, proliferation, and metastasis [16]. For example, Dror et al. (2016) reported that melanosomal miR-211, which is secreted by melanoma cells and absorbed by fibroblasts, can activate the mitogen-activated protein kinase (MAPK) signaling pathway and induce fibroblast reprogramming into CAFs [17]. Our previous study showed that melanoma cell-secreted microvesicles can induce the transformation of normal fibroblasts into CAFs [6]. Here, our results indicate that melanoma cell-released exosomes can elevate α-SMA and FAP expression in NIH/3T3 cells, indicating that melanoma cell-released exosomes can also trigger normal fibroblast reprogramming into CAFs. Interestingly, exosomes secreted by human melanoma cell line A375 can also induce HGF transforming into CAFs. These results collectively indicate that exosomes play a crucial role in CAF transformation. Yasushi Kojima et al. (2010) reported that the establishment of the self-sustaining TGF-β and SDF-1 autocrine-signaling loops initiate and maintain the differentiation of fibroblasts into tumor-promoting CAFs [18]. CAFs have been reported to promote tumor angiogenesis. In our study, CAFs exhibited upregulated expression and secretion of the angiogenic factors VEGFa, FGF2, and MMP9. The CM of CAFs promoted EC proliferation, migration, and tube formation. In the Matrigel plug assay, CAF groups exhibited high MVD. These results indicate that melanoma cell-secreted exosomes can induce and enhance the proangiogenic capability of CAFs in the tumor microenvironment. VEGFa and FGF2 are two potent proangiogenic factors. Binding to VEGFa and FGF2 results in the downstream activation of various signaling pathways in ECs; this condition can promote EC proliferation, survival, and migration. In melanoma patients, elevated serum levels of VEGF, FGF2, and other soluble proangiogenic factors have been demonstrated and are closely correlated with poor clinical outcome [19]. Increased VEGF and FGF2 expression and accumulation were identified in the tumor microenvironment. MMP9 can also release VEGF from the extracellular matrix (ECM) [20]. By secreting proangiogenic ECM remodeling enzymes such as MMP9, CAFs reconstruct the ECM to facilitate ECs to cross this structural barrier. Therefore, our results confirm that CAFs play an important role in tumor angiogenesis.

Accumulating evidence have shown the strong proangiogenic effects of JAK/STAT signaling pathway [21–26]. JAK2/STAT3 pathway has been reported to regulate several critical proangiogenic factors, such as VEGFa, MMP-2, MMP-9, insulin-like growth factor 1 (IGF-1), and FGF2 [11]. However, whether the JAK2/STAT3 signaling pathway participates in the proangiogenic switch of CAFs in melanoma microenvironment has not been reported. In our study, treatment with melanoma cell-secreted exosomes significantly elevated the phosphorylation levels of JAK2 and STAT3 in CAFs. When the JAK2/STAT3 signaling pathway was blocked by AG490, the expressions of VEGFa, FGF2, and MMP9 reduced significantly. In addition, the proangiogenic effect of the CM of CAFs was also remarkably suppressed by AG490. These results collectively indicate that the JAK2/STAT3 signaling pathway can regulate the expressions of key proangiogenic factors (VEGFa, FGF2, and MMP9) and plays a critical role in the proangiogenic switch of CAFs.

Several studies indicated SOCS1 as a tumor suppressor gene. Aberrant methylation of SOCS1 silences SOCS1 and activates the JAK2/STAT3 pathway in hepatocellular carcinoma cells (HCC), resulting in the development of HCC [27]. A previous report from Fengju Huang et al. (2008) indicated that SOCS1 expression is reduced in brain metastasis of melanoma. The loss of SOCS1 expression leads to the activation of the JAK2/STAT3 signaling pathway and overexpression of MMP-2, FGF2, and VEGF and enhanced invasion and angiogenesis of melanoma cells, consequently promoting brain metastasis [13]. Previous studies have focused on the relationship between SOCS and the JAK/STAT signaling pathway. However, the reason for decreased SOCS1 expression in tumor tissues remains incompletely understood. Here, we observed that melanoma cell-secreted exosomal miR-155 suppressed SOCS1 expression in CAFs. Suppression of SOCS1 in CAFs activated the JAK2/STAT3 signaling pathway and then promoted the expressions of MMP-9, FGF2, and VEGFa, triggering the proangiogenic switch of CAFs.

MiR-155 have been reported to play an important role in promotion of inflammation [28, 29], regulation of adipose tissue function [30], modulation of glucose homeostasis [31]. Exosome-delivered miR-155 can also be taken up by recipient cells and modulate several responses in these recipient cells [32]. Silencing of miR-155 by using antisense oligomers (antimiRs) as anti-cancer drug is an evolving therapeutic strategy [33]. Overexpression of miR-155 is identified in primary melanoma and increases in melanoma exhibiting regional progression [34–36]. Consistently, our study showed the elevated miR-155 expression in highly metastatic (B16F10) compared with weakly metastatic (B16) melanoma cell lines. A relatively high levels of miR-155 was detected in B16F10-released exosomes compared with exosomes extracted from B16 cells. Previous studies indicated that overexpression of miR-155 can promote melanoma cell proliferation and invasion [37]. However, the effects of overexpression of miR-155 on melanoma angiogenesis have not been reported. Our study showed that miR-155 can be transferred into CAFs by melanoma cell-secreted exosomes. Elevated levels of miR-155 in B16-secreted exosomes can suppress the expression of SOCS1; upregulate the phosphorylation levels of JAK2 and STAT3; enhance the expressions of MMP-9, FGF2, and VEGFa; and enhance the promotive effect of CAFs on EC proliferation, migration, and tube formation. Reversely, the decreased levels of miR-155 in B16F10-secreted exosomes exhibit upregulated expression of SOCS1; suppress the activation of JAK2/STAT3 signaling pathway; downregulate the expressions of MMP-9, FGF2, and VEGFa; and alleviate the promotive effect of CAFs on EC proliferation, migration, and tube formation. In vivo xenograft models, elevated levels of miR-155 in B16-secreted exosomes significantly increased the MVD of xenografts, but decreased levels of miR-155 in B16F10-secreted exosomes lowered the MVD of xenografts. Our results suggest that exosomal miR-155 plays an important role in the proangiogenic switch of CAFs via SOCS1/JAK2/STAT3 signaling pathway. However, inhibiting miR-155 in melanoma cell-secreted exosomes cannot reduce the proangiogenic factor expression to the original level, suggesting that additional factors in melanoma cell-secreted exosomes can be involved in the proangiogenic switch of CAFs and require further investigation. Further investigations should determine whether treatment with melanoma cell-secreted exosomes promotes primary miR-155 to be processed via the intermediate precursor miR-155 to the functional mature miR-155 in CAFs. In our study, the xenografts with NIH/3T3 treated with exosomes containing elevated levels of miR-155 exhibited a large tumor volume and were heavy (Fig. 7c, d). Conversely, the xenografts with NIH/3T3 treated with exosomes containing lowered levels of miR-155 exhibited small tumor volume and were light (Fig. 7i, j). Therefore, additional studies should also examine whether miR-155 overexpression can enhance the effects of CAFs on melanoma proliferation and metastasis.

## Conclusions

In conclusion, our study demonstrates that melanoma cell-secreted exosomal miR-155 can trigger normal fibroblast reprogramming into proangiogenic CAFs by decreasing SOCS1, to activate JAK2/STAT3 signaling pathway. CAFs exhibit increasing expressions and secretion of VEGFa, FGF2, and MMP9, promoting proliferation, migration, and tube formation of ECs, and melanoma angiogenesis. Our study elucidates a new molecular mechanism underlying the crosstalk between melanoma cells and fibroblasts to promote tumor angiogenesis, which can contribute to efficient prevention and therapeutic strategies for melanoma.

## Additional file

Additional file 1: Supplementary figures and figure legends. (DOCX 1255 kb)

## Abbreviations

3′UTR: 3′ untranslated region
anti-miR-155: miR-155 inhibitor
anti-NC: inhibitor negative control
BCA: bicinchoninic acid
CAFs: cancer-associated fibroblasts
CCK-8: Cell Counting Kit-8
CM: conditioned medium
Ct: cycle threshold
DAPI: 4′6-diamidino-2-phenylindole
DMEM: Dulbecco’s Modified Eagle’s Medium
EC: endothelial cell
ECM: extracellular matrix
ELISA: Enzyme-linked immunosorbent assay
EVs: extracellular vesicles
FAP: fibroblast activation protein
FBS: fetal bovine serum
FGF2: fibroblast growth factor 2
FITC: fluorescein isothiocyanate
GAPDH: glyceraldehyde 3-phosphate dehydrogenase
HGFs: human gingival fibroblasts
Hsp90: Heat shock protein 90
IGF-1: insulin-like growth factor 1
JAK2: Janus kinase 2
MAPK: mitogen-activated protein kinase
miR-155: mmu-miR-155-5p
miRNAs: microRNAs
miR-NC: miR-mimic negative control
MMP9: matrix metalloproteinase 9
MVD: microvessel density
PBS: phosphate buffer solution
P-JAK2: phospho-JAK2
P-STAT3: phospho-STAT3
PVDF: polyvinylidene difluoride
RPMI: Roswell Park Memorial Institute
SEM: standard error of the mean
SOCS1: suppressor of cytokine signaling 1
SPSS: Statistical Product and Service Solutions
STAT3: signal transducer and activator of transcription 3
TBST: Tris-buffered saline containing 0.05% Tween 20
Tsg101: tumor susceptibility gene 101
VEGFa: vascular endothelial growth factor A
α-SMA: α-smooth muscle actin
