# Supplementary material for: Melanoma cell-secreted exosomal miR-155-5p induce proangiogenic switch of cancer-associated fibroblasts via SOCS1/JAK2/STAT3 signaling pathway
Source: J Exp Clin Cancer Res. 2018 Oct 3;37:242. doi: 10.1186/s13046-018-0911-3 (PMC6169013; doi:10.1186/s13046-018-0911-3)
Supplement: Supplementary file 1 — Supplementary figures and figure legends. (DOCX 1255 kb) [file 13046_2018_911_MOESM1_ESM.docx]

**Melanoma cell-secreted exosomal miR-155-5p induce proangiogenic switch of cancer-associated fibroblasts via SOCS1/JAK2/STAT3 signaling pathway.**

Xiaocheng Zhou^1^, Tinglin Yan^1^, Chunming Huang^2^, Zhi Xu^3^, Lin Wang^1^, Erhui Jiang^1^, Hui Wang^1^, Yang Chen^1^, Ke Liu^1,4^, Zhe Shao^1,4^, and Zhengjun Shang^1,4,*^

1. The State Key Laboratory Breeding Base of Basic Science of Stomatology (Hubei-MOST) & Key Laboratory for Oral Biomedicine Ministry of Education, Wuhan University, Wuhan, China
2. Center of Stomatology, Tongji Hospital, Tongji Medical College, Huazhong University of Science and Technology, Wuhan, China
3. Department of Stomatology, Union Hospital, Tongji Medical College, Huazhong University of Science and Technology, Wuhan, China
4. Department of Oral and Maxillofacial-Head and Neck Oncology, School and Hospital of Stomatology, Wuhan University, Wuhan, China

*Corresponding author: Zhengjun Shang, tel.: +86-27-8768-6129, fax: +86-27-8787-3260, email: shangzhengjun@whu.edu.cn

**E-mail:** Xiaocheng Zhou ([2014203040019@whu.edu.cn](mailto:2014203040019@whu.edu.cn)), Tinglin Yan ([yantinglin890929@hotmail.com](mailto:yantinglin890929@hotmail.com)), Chunming Huang ([huangchunming89@whu.edu.cn](mailto:huangchunming89@whu.edu.cn)), Zhi Xu ([2017xh0090@hust.edu.cn](mailto:2017xh0090@hust.edu.cn)), Lin Wang ([2013283040056@whu.edu.cn](mailto:2013283040056@whu.edu.cn)), Erhui Jiang ([jiangerhui@whu.edu.cn](mailto:jiangerhui@whu.edu.cn)), Hui Wang ([2017203040017@whu.edu.cn](mailto:2017203040017@whu.edu.cn)), Yang Chen ([2017203040011@whu.edu.cn](mailto:2017203040011@whu.edu.cn)), Ke Liu ([liuke.1999@whu.edu.cn](mailto:liuke.1999@whu.edu.cn)), Zhe Shao (
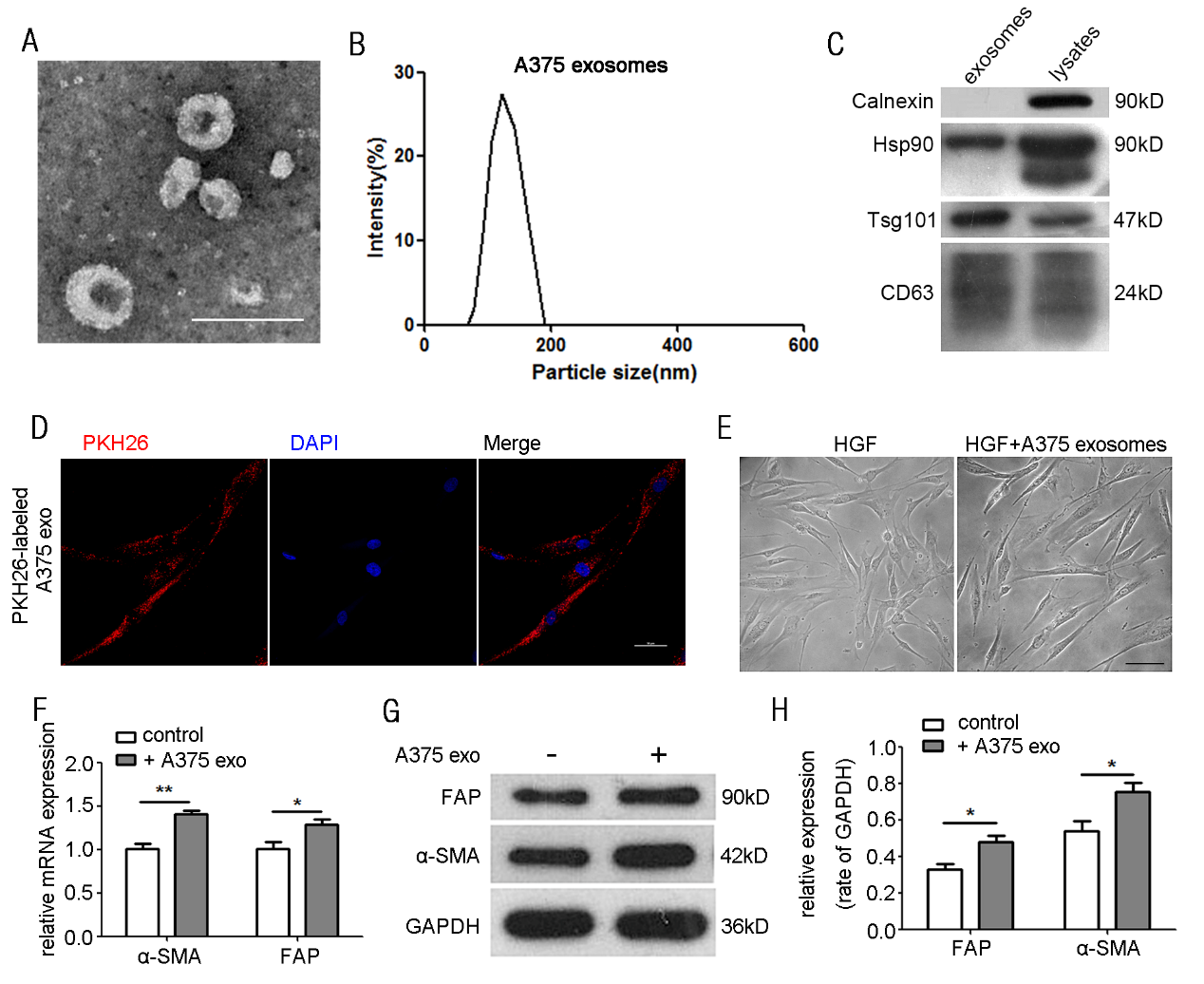
[shaozhe@whu.edu.cn).
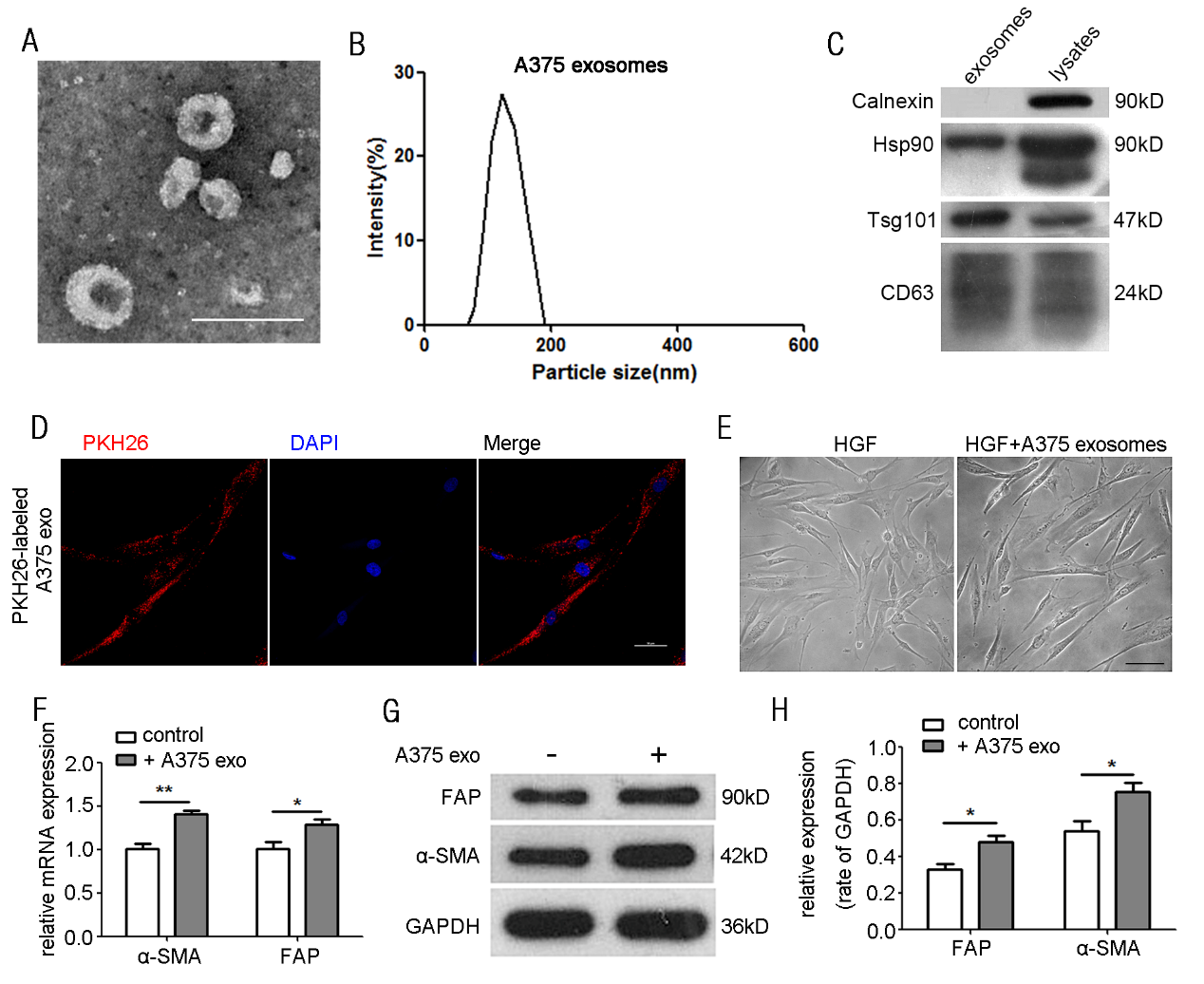
](mailto:shaozhe@whu.edu.cn).Additional)**[Additional](mailto:shaozhe@whu.edu.cn).Additional) file 1: Figure S1. (A, B)** Exosomes released by A375 cells were identified by using transmission electron microscope and dynamic light scattering analysis. Scale bar, 200 nm. **(C)** Characterization of exosomes with Western blot analysis. Exosomal markers: Hsp90, Tsg101, and CD63. Calnexin served as a cellular contaminants marker. Cell lysates of A375 was used as control. **(D)** Confocal microscope images showed the uptake of PKH26-labeled exosomes in HGF cells. Scale bar, 50 μm. **(E)** The phase morphology of HGF cells with different treatments. Scale bar, 50 μm. **(F)** The expressions of α-SMA and FAP were investigated by RT-PCR after HGF cells were treated with exosomes (20 μg/mL) extracted from A375 cells for 24 h. GAPDH was used as the normalization control. **(G, H)** The expressions of α-SMA and FAP in HGF cells with different treatments were evaluated by Western blot and densitometry analysis. Independent experiments performed in triplicate. * *P* < 0.05, ** *P* < 0.01 vs. control group. Values are represented as means ± SEM. DAPI: 4'6-diamidino-2-phenylindole. Exo: exosomes. FAP: fibroblast activation protein. GAPDH: Glyceraldehyde 3-phosphate dehydrogenase. HGF: human gingival fibroblast. Hsp90: heat shock protein 90. SEM: standard error of the mean. Tsg101: tumor susceptibility gene 101. α-SMA: α-smooth muscle actin.


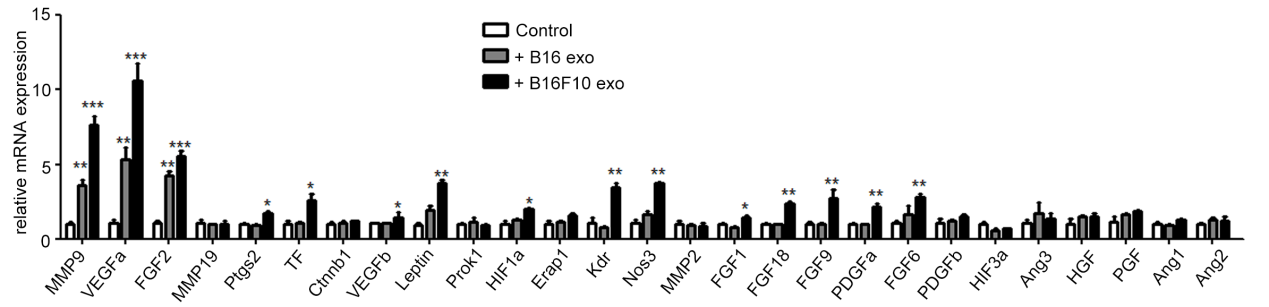


**Additional file 1: Figure S2.** Treatment with B16- and B16F10-secreted exosomes for 24 h regulated the expressions of proangiogenic factors in NIH/3T3 cells as shown by RT-PCR. * *P* < 0.05, ** *P* < 0.01, *** *P* < 0.001. Independent experiments performed in triplicate. Values are expressed as means ± SEM. Student’s t-tests. Exo: exosomes. SEM: standard error of the mean.


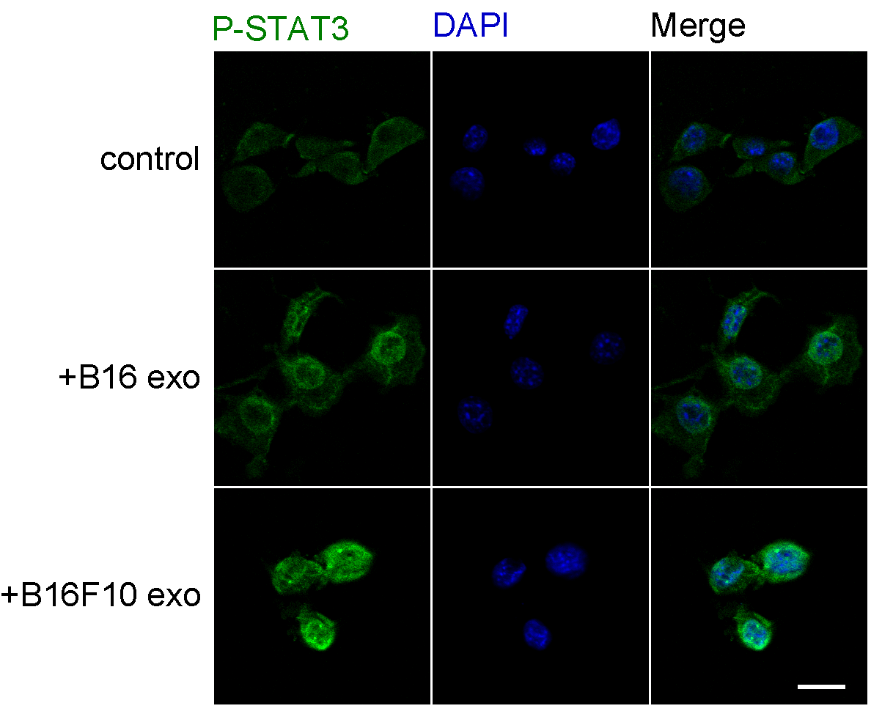


**Additional file 1: Figure S3.** The nuclear localization of P-STAT3 in NIH/3T3 cells was examined by confocal microscope after treatment with B16- and B16F10-secreted exosomes for 6 h. These images are representative images of triplicate independent experiments. Scale bar, 50 μm. DAPI: 4'6-diamidino-2-phenylindole. Exo: exosomes.


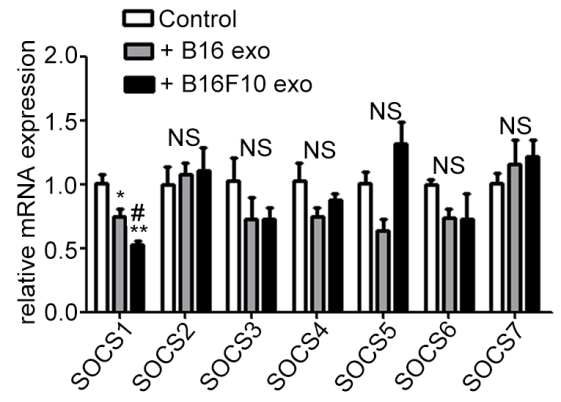


**Additional file 1: Figure S4.** The expressions of SOCS family were investigated by RT-PCR after NIH/3T3 cells were treated with exosomes (20 μg/mL) extracted from B16 and B16F10 cells for 24 h. GAPDH was used as the normalization control. * *P* < 0.05, ** *P* < 0.01 vs. control group. # *P* < 0.05 vs. NIH/3T3 cells treated with B16-secreted exosomes. Independent experiments performed in triplicate. Values are expressed as means ± SEM. Exo: exosomes. GAPDH: Glyceraldehyde 3-phosphate dehydrogenase. NS: no statistical differences. SOCS: suppressor of cytokine signaling.


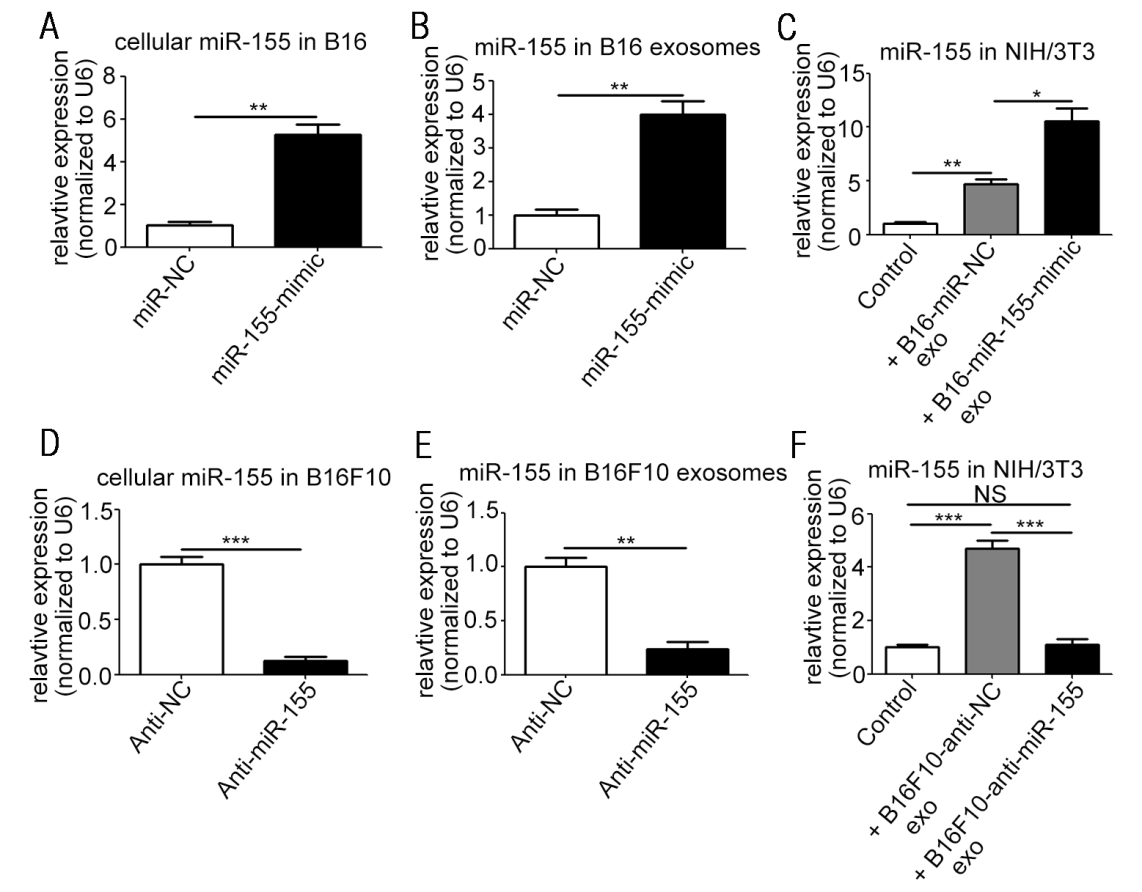


**Additional file 1: Figure S5. (A, B)** Transfection of miR-155-mimic in B16 cells elevated the expression level of miR-155 in B16 cells and B16-secreted exosomes. **(C)** The expression levels of miR-155 in NIH/3T3 cells after treatment of exosomes from miR-NC-transfected and miR-155-mimic-transfected B16 cells. **(D, E)** Transfection of anti-miR-155 in B16F10 cells downregulated the expression level of miR-155 in B16F10 cells and B16F10-secreted exosomes. **(F)** The expression levels of miR-155 in NIH/3T3 cells after treatment of exosomes from anti-NC-transfected and anti-miR-155-transfected B16F10 cells. * *P* < 0.05, ** *P* < 0.01, *** *P* < 0.001. Independent experiments performed in triplicate. Values are expressed as means ± SEM. Student’s t-tests. Anti-NC: inhibitor negative control. Anti-miR-155: miR-155 inhibitor. Exo: exosomes. MiR-NC: miR-negative control. NS: no statistical differences. SEM: standard error of the mean.

**Additional file 1: Table S1.** Sequences of the synthesized oligonucleotides and the primers.

| **name** | **Sequences** | | |
| --- | --- | --- | --- |
|  | **Sense (5’-3’)** | | **Antisense (5’-3’)** |
| miR-negative control (miR-NC) | UUCUCCGAACGUGUCACGUTT | | ACGUGACACGUUCGGAGAATT |
| mmu-miR-155-5p mimic  (miR-155 mimic) | UUAAUGCUAAUUGUGAUAGGGGU | | CCCUAUCACGAUUAGCAUUAAUU |
| inhibitor negative control (anti-NC) | CAGUACUUUUGUGUAGUACAA | | |
| mmu-miR-155-5p inhibitor  (anti-miR-155) | ACCCCUAUCACAAUUAGCAUUAA | | |
| Mmu-miR-155-5p | UUAAUGCUAAUUGUGAUAGGGGU | | |
| α-SMA | CCCAGACATCAGGGAGTAATGG | TCTATCGGATACTTCAGCGTCA | |
| FAP | GCGACATACTACATCTACGACCT | AGATACTGAATTGGACGAGGGAG | |
| FGF2 | GCGACCCACACGTCAAACTA | CCGTCCATCTTCCTTCATAGC | |
| VEGFa | GCACATAGAGAGAATGAGCTTCC | CTCCGCTCTGAACAAGGCT | |
| MMP9 | GCAGAGGCATACTTGTACCG | TGATGTTATGATGGTCCCACTTG | |
| GAPDH | TGACCTCAACTACATGGTCTACA | CTTCCCATTCTCGGCCTTG | |
| SOCS1 | CTGCGGCTTCTATTGGGGAC | AAAAGGCAGTCGAAGGTCTCG | |
| SOCS2 | AGTTCGCATTCAGACTACCTACT | TGGTACTCAATCCGCAGGTTAG | |
| SOCS3 | ATGGTCACCCACAGCAAGTTT | TCCAGTAGAATCCGCTCTCCT | |
| SOCS4 | ACGACACACTGTTCCTATGAGT | TTGGCAGTTATGACACAAGGC | |
| SOCS5 | GAACCCCAACAGATGTCCGTC | GGATCTCTGCGGCACAGTTTT | |
| SOCS6 | AAGCAAAGACGAAACTGAGTTCA | CAGCTCCCGAATAAAGAGTCATC | |
| SOCS7 | GAAACCCAGGTTGACAAGAACT | TCCACAAGCGATACTGTCTCA | |
| MMP19 | CCTGGTCCCATGCCAAACC | CCCTTGAAAGCATAAGTCTTCCC | |
| Ptgs2 | TTCAACACACTCTATCACTGGC | AGAAGCGTTTGCGGTACTCAT | |
| TF | CTGGAAAAACAAGTGCTTCTCG | ACAGAGAGGACCTTTGCTTCA | |
| Ctnnb1 | TCCCATCCACGCAGTTTGAC | TCCTCATCGTTTAGCAGTTTTGT | |
| VEGFb | GCCAGACAGGGTTGCCATAC | GGAGTGGGATGGATGATGTCAG | |
| Leptin | GAGACCCCTGTGTCGGTTC | CTGCGTGTGTGAAATGTCATTG | |
| Prok1 | CATCATGCTCCTTCTAGCAACG | CGCAGCCACAGACTGATAGC | |
| HIF1a | ACCTTCATCGGAAACTCCAAAG | CTGTTAGGCTGGGAAAAGTTAGG | |
| Erap1 | TAATGGAGACTCATTCCCTTGGA | TGCTGAGGTTTGCATGGATCA | |
| Kdr | CTGGAGCCTACAAGTGCTCG | GAGGTTTGAAATCGACCCTCG | |
| Nos3 | CGAAGCGTGTGAAGGCAAC | TTGTACGGGCCTGACATTTCC | |
| MMP2 | ACCTGAACACTTTCTATGGCTG | CTTCCGCATGGTCTCGATG | |
| FGF1 | CCCTGACCGAGAGGTTCAAC | GTCCCTTGTCCCATCCACG | |
| FGF18 | CCTGCACTTGCCTGTGTTTAC | TGCTTCCGACTCACATCATCT | |
| FGF9 | ATGGCTCCCTTAGGTGAAGTT | TCCGCCTGAGAATCCCCTTT | |
| PDGFa | TGGCTCGAAGTCAGATCCACA | TTCTCGGGCACATGGTTAATG | |
| FGF6 | CAGGCTCTCGTCTTCTTAGGC | AATAGCCGCTTTCCCAATTCA | |
| PDGFb | TGCTGCACAGAGACTCCGTA | GATGAGCTTTCCAACTCGACTC | |
| HIF3a | GAAGTTCACATACTGCGACGA | GTCCAAAGCGTGGATGTATTCAT | |
| Ang3 | TGAGTCTGGATGTGATCCCTC | GACCTGGAATCGAGAATTGCTT | |
| HGF | AAAGGGACGGTATCCATCACT | GCGATAGCTCGAAGGCAAAAAG | |
| PGF | TCTGCTGGGAACAACTCAACA | GTGAGACACCTCATCAGGGTAT | |
| Ang1 | CCAGGCCCGTTGTTCTTGAT | GCAAACCATTCTCACAGGCAATA | |
| Ang2 | AGAATAAGCAAGTCTCGCTTCC | TGAACCCTTTAGAGGCTCGGT | |
